# A novel technique for biliary stent retrieval using a sphincterotome and drill dilator during balloon-assisted endoscopic retrograde cholangiopancreatography

**DOI:** 10.1055/a-2900-8776

**Published:** 2026-07-21

**Authors:** Kosuke Takahashi, Eisuke Ozawa, Mizuki Kitagawa, Masashi Shibata, Hisamitsu Miyaaki

**Affiliations:** 1 Department of Gastroenterology and Hepatology 200674 Nagasaki University Graduate School of Biomedical SciencesNagasakiNagasakiJapan

**Fig. 1:**
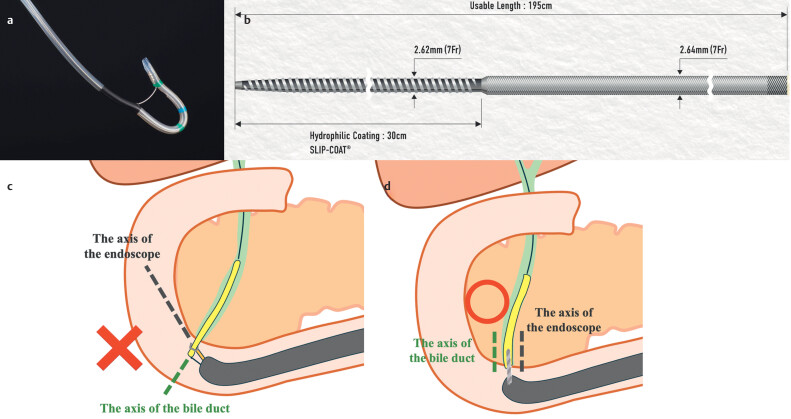
( **a** ) A novel sphincterotome with high rotational performance, allowing a flexible blade orientation. ( **b** ) A novel drill dilator. ( **c** ) In BE-ERCP, the stent retrieval using conventional devices may be difficult because of limitations related to the narrow working channel and poor coaxial alignment. ( **d** ) Using the novel sphincterotome, a guidewire is successfully advanced into the biliary stent; the drill dilator then firmly engages within the stent lumen, facilitating coaxial alignment between the stent and endoscope and enabling easier stent removal. BE-ERCP, balloon-assisted endoscopic retrograde cholangiography.

Balloon-assisted endoscopic retrograde cholangiopancreatography (BE-ERCP) is technically challenging in patients with surgically altered anatomy because achieving coaxial alignment with the bile duct is often difficult. 1​ In addition, the narrow working channel limits device maneuverability and complicates stent retrieval. We report successful biliary stent retrieval using a novel sphincterotome and drill dilator in a patient with malignant biliary obstruction and tumor-induced papillary displacement ( Video 1 ; Fig. 1a–d ).

**Video 1** A novel technique for biliary stent retrieval using a sphincterotome and drill dilator during balloon-assisted endoscopic retrograde cholangiopancreatography.


An 81-year-old woman with a history of total gastrectomy with Roux-en-Y reconstruction presented with obstructive jaundice caused by ampullary carcinoma. After failed BE-ERCP, percutaneous transhepatic biliary drainage was performed, followed by biliary stenting using the rendezvous technique. Subsequent stent occlusion necessitated stent exchange.

**Fig. 2:**
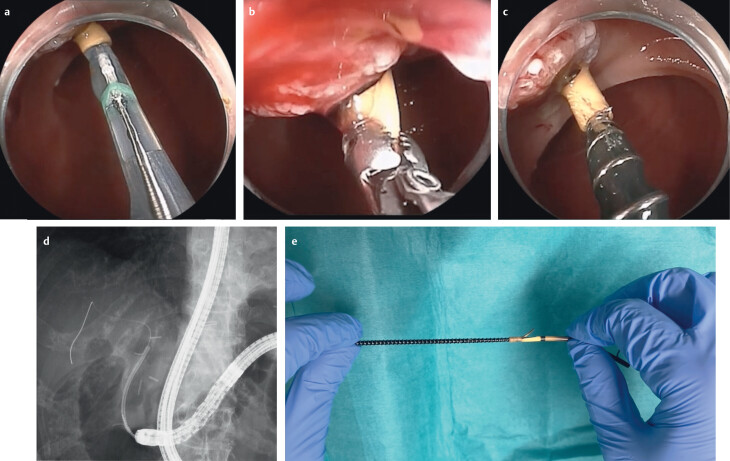
( **a** ) An endoscopic view showing successful biliary cannulation using a novel sphincterotome with high rotational performance, enabling precise blade orientation. ( **b** ) An endoscopic view showing unsuccessful stent retrieval using grasping forceps owing to poor coaxial alignment. ( **c** ) An endoscopic view showing the advancement of the drill dilator to the distal end of the stent with clockwise rotation, allowing firm engagement within the stent lumen. ( **d** ) A fluoroscopic image demonstrating restoration of coaxial alignment and successful stent retrieval while maintaining guidewire access. ( **e** ) The retrieved stent and drill dilator showing strong adhesion.

After reaching the papilla, retroflexion failed to achieve coaxial alignment with the bile duct, and guidewire insertion alongside the existing stent was difficult. Therefore, a novel sphincterotome with high rotational performance and dual-action functionality was used, enabling the precise blade orientation and successful biliary cannulation ( Fig. 2a ). Stent retrieval using rotational grasping forceps was unsuccessful because of poor coaxial alignment between the endoscope and the stent ( Fig. 2b ). A novel drill dilator was advanced into the distal end of the stent and rotated clockwise, firmly engaging the stent lumen ( Fig. 2c–e ). This maneuver improved coaxial alignment and enabled easy stent retrieval while maintaining guidewire access. A covered metallic stent was subsequently placed. No intra- or post-procedural adverse events occurred. Clinical improvement was achieved without recurrence of cholangitis.

Although drill dilators have been used for migrated stent retrieval during conventional ERCP, 2​ 3​ 4​ ​5​ their use during BE-ERCP has not been reported. The combined use of a novel sphincterotome and drill dilator may represent an effective troubleshooting technique for difficult stent retrieval during BE-ERCP.

Endoscopy_UCTN_Code_TTT_1AR
